# Association of cardiac rehabilitation and health-related quality of life following acute myocardial infarction

**DOI:** 10.1136/heartjnl-2020-316920

**Published:** 2020-08-21

**Authors:** Ben Hurdus, Theresa Munyombwe, Tatendashe Bernadette Dondo, Suleman Aktaa, Gerrard Oliver, Marlous Hall, Patrick Doherty, Alistair S Hall, Chris P Gale

**Affiliations:** 1 Leeds Institute for Data Analytics, Leeds Institute of Cardiovascular and Metabolic Medicine, Leeds, UK; 2 Department of Cardiology, Leeds Teaching Hospitals NHS Trust, Leeds, UK; 3 Patient Collaborator, Lancashire, UK; 4 Health Sciences, University of York, York, North Yorkshire, UK

**Keywords:** cardiac rehabilitation, cardiac risk factors and prevention, acute coronary syndromes, quality and outcomes of care

## Abstract

**Objective:**

To study the association of cardiac rehabilitation and physical activity with temporal changes in health-related quality of life (HRQoL) following acute myocardial infarction (AMI).

**Methods:**

Evaluation of the Methods and Management of Acute Coronary Events-3 is a nationwide longitudinal prospective cohort study of 4570 patients admitted with an AMI between 1 November 2011 and 17 September 2013. HRQoL was estimated using EuroQol 5-Dimension-3 Level Questionnaire at hospitalisation, 30 days, and 6 and 12 months following hospital discharge. The association of cardiac rehabilitation and self-reported physical activity on temporal changes in HRQoL was quantified using inverse probability of treatment weighting propensity score and multilevel regression analyses.

**Results:**

Cardiac rehabilitation attendees had higher HRQoL scores than non-attendees at 30 days (mean EuroQol 5-Visual Analogue Scale (EQ-VAS) scores: 71.0 (SD 16.8) vs 68.6 (SD 19.8)), 6 months (76.0 (SD 16.4) vs 70.2 (SD 19.0)) and 12 months (76.9 (SD 16.8) vs 70.4 (SD 20.4)). Attendees who were physically active ≥150 min/week had higher HRQoL scores compared with those who only attended cardiac rehabilitation at 30 days (mean EQ-VAS scores: 79.3 (SD 14.6) vs 70.2 (SD 17.0)), 6 months (82.2 (SD 13.9) vs 74.9 (SD 16.7)) and 12 months (84.1 (SD 12.1) vs 75.6 (SD 17.0)). Cardiac rehabilitation and self-reported physical activity of ≥150 min/week were each positively associated with temporal improvements in HRQoL (coefficient: 2.12 (95% CI 0.68 to 3.55) and 4.75 (95% CI 3.16 to 6.34), respectively).

**Conclusions:**

Cardiac rehabilitation was independently associated with temporal improvements in HRQoL at up to 12 months following hospitalisation, with such changes further improved in patients who were physically active.

## Introduction

Health-related quality of life (HRQoL) is an important outcome measure following acute myocardial infarction (AMI).^1^ Healthcare professionals have historically focused on objective measures of poor health, such as mortality and life expectancy, but often patients consider improvements to HRQoL equally important to length of life.^2^ The benefits of exercise post-AMI on mortality have been demonstrated since the early 1950s, yet only recently has its potential to enhance HRQoL been recognised in this group.^3 4^

While cardiac rehabilitation has been associated with better HRQoL in patients following AMI,^3^ the majority of studies are limited by small sample sizes, cross-sectional designs, have limited generalisability or have not studied repeated (longitudinal) measures of HRQoL in populations.^5–7^ Moreover, the literature suggests that cardiac rehabilitation is an underused intervention with referral rates reported at around 50% and uptake frequently lower than this.^8 9^

The Evaluation of the Methods and Management of Acute Coronary Events (EMMACE)-3 is a national longitudinal prospective cohort study that collected data pertaining to the referral and uptake of cardiac rehabilitation in the National Health Service (NHS) of England. Among other patient-level items, it collected HRQoL scores, self-reported physical activity status, comorbidities, treatments and clinical outcomes for patients who were admitted to 48 hospitals between 1 November 2011 and 17 September 2013 with an acute coronary syndrome.^10^ This study aimed to investigate the association of cardiac rehabilitation and temporal changes in HRQoL estimated at multiple time points and to explore the impact of self-reported physical activity status on HRQoL trajectories.

## Methods

### Setting and design

The study was based on data from 4570 patients who participated in EMMACE-3, a national longitudinal cohort study.^10^ Eligible patients included all adults aged 18 years and over hospitalised with AMI (ST-elevation myocardial infarction (STEMI) or non-ST-elevation myocardial infarction (NSTEMI)) who were admitted to 48 NHS hospitals in England between 1 November 2011 and 17 September 2013. Records for consenting patients were linked to the UK Myocardial Ischaemia National Audit Project (MINAP) to obtain data about their medical history, comorbidities, cardiac biomarkers, type of AMI (STEMI or NSTEMI), hospital treatments and discharge medications.^11^ The EMMACE-3 study protocol, of which this research is a subset, is available online (https://bmjopen.bmj.com/content/5/6/e006256).^10^

### Assessment of HRQoL

The primary outcome, self-reported HRQoL, was quantified using EuroQol 5-Dimension-3 Level Questionnaire (EQ-5D-3L), which has been validated in patients with AMI^12^ and consists of two component parts, a descriptive classification (EuroQol 5-Dimension (EQ-5D)) and a visual analogue scale (EuroQol 5-Visual Analogue Scale (EQ-VAS)). EQ-5D comprises five dimensions: mobility, self-care, usual activities, pain/discomfort and anxiety/depression, with each dimension subdivided into three levels: no problems, some problems, extreme problems, indicating a patient’s perceived level of function. Each level carries a weighted score which is combined across the five dimensions to total a single index score, which is then standardised to the UK population.^13^ For the purposes of this study, we combined the levels ‘some problems’ and ‘extreme problems’ so that responses were binary. EQ-5D scores ranged from −0.5 to 1.0, with negative scores indicating states ‘worse than death’, 0 indicating no quality of life or ‘death’, and 1 indicating full health.^14^ EQ-VAS is an analogue scale of 0–100 in which participants are required to indicate their own perceived health, with 0 indicating ‘worst imaginable health state’ and 100 indicating ‘best imaginable health state’.^15^ A difference in a score of 0.05 for EQ-5D and 7 for VAS is regarded as clinically important.^16^ For EMMACE-3, EQ-5D-3L data were collected at hospitalisation and at 30 days, and 6 and 12 months following discharge from the hospital.

### Statistical analyses

Baseline characteristics for categorical data were described using frequencies and proportions. Normally distributed continuous data were described using means and SD, and non-normally distributed data were described using medians and IQRs. The differences in baseline characteristics between STEMI and NSTEMI were summarised using t-tests, χ^2^ tests, and Wilcoxon rank-sum tests appropriate to the data type and distribution.

Propensity score-based weighting coupled with a multilevel linear regression model (repeated measurements nested within patients and patients nested within hospitals) was applied to assess the relationship of attending cardiac rehabilitation on HRQoL.^17^ Briefly, the modelling involved a two-step approach: (1) a treatment assignment model estimating the propensity of attending cardiac rehabilitation at 30 days following discharge from the hospital was used to derive inverse-probability weights in order to balance any systematic differences in baseline characteristics between cardiac rehabilitation attendees and non-attendees (see online supplementary section 1 and 2) and (2) an outcome model assessing the impact of cardiac rehabilitation on HRQoL following AMI using a multilevel linear regression fitted to the weighted data at 30 days and 6 and 12 months.

10.1136/heartjnl-2020-316920.supp1 Supplementary data

Since the weighting was conducted using covariates observed at baseline, longitudinal multilevel regression models were additionally adjusted for the following patient-level factors in order to minimise potential residual confounding: baseline EQ-VAS, AMI phenotype (STEMI vs NSTEMI), sex, age, Body Mass Index (BMI), Index of Multiple Deprivation (IMD) score, smoking status (never vs current or ex-smoker), family history of coronary heart disease (CHD), previous angina, history of diabetes mellitus, hypertension, heart failure, peripheral vascular disease, cerebrovascular disease, chronic renal failure, chronic obstructive pulmonary disease (COPD) or asthma, hypercholesterolaemia, previous percutaneous coronary intervention (PCI), previous coronary artery bypass graft (CABG) surgery, previous AMI, PCI during AMI hospitalisation, CABG surgery during AMI hospitalisation, reinfarction during index AMI hospitalisation, medications prescribed at discharge from the hospital (including aspirin, beta-blockers, statins and ACE inhibitors) and self-reported physical activity status. Missing data were imputed using multiple imputation by chained equations (10 datasets from 20 iterations), and a default imputation strategy based on clinical expert opinion was implemented for selected treatment variables (online supplementary section 2). Pooled estimates and accompanying 95% CIs for each model were generated according to Rubin’s rules.^18^ Analysis were performed using STATA MP64 V.14 (StataCorp, www.stata.com), and p values of <0.05 were considered statistically significant.

### Patient involvement

While no patients were involved in setting the research question or the study design, the work is coauthored by a patient representative (GO) who helped with the interpretation of the findings and provided a critical review of the manuscript text.

## Results

### Study sample

From 5557 hospitalisations of consented patients across 48 hospitals, 181 (3.3%) patients withdrew from the study; we excluded 510 (9.2%) due to failed data linkage to MINAP and 296 (5.3%) who did not have a discharge diagnosis of AMI, leaving an analytical cohort of 4570 (online supplementary eFigure 1). For the weighted multilevel linear regression model, we analysed an effective sample size of 3438 because a further 1132 patients were excluded due to missing exposure/outcome data. Questionnaire response rates were 96.4% (4403/4570), 74.3% (3395/4570), 65.1% (2973/4570) and 61.9% (2828/4570) at hospitalisation, 30 days, and 6 and 12 months, respectively. Sixteen patients (0.3%) died in the hospital. Missing data levels were <7% for baseline patient demographic characteristics, except for IMD (5.8%), BMI (39.3%), family history of CHD (13.1%) and ethnicity (22.0%) (table 1). The mean age for the analytical cohort was 63.6 (SD 11.9) years; 25.2% were women; 76.3% were Caucasian; the median BMI was 27.8 (25.1–31.3) kg/m^2^; the median IMD score was 18.5 (IQR 11.0–31.8); and 65.9% were current or ex-smokers. Comorbidity was common, including hypertension (42.7%), angina (19.6%), diabetes mellitus (15.9%) and COPD or asthma (12.1%) (table 1).

**Table 1:** Patient baseline characteristics stratified by type of acute myocardial infarction (STEMI vs NSTEMI)

STEMI, n=1856	NSTEMI, n=2714	P value	All AMI, n=4570	Missing, n (%)
Variables					
Age (years), mean (SD)	60.9 (11.5)	65.5 (11.9)	<0.001	63.6 (11.9)	17 (0.4)
Women, n (%)	432 (23.3)	720 (26.6)	0.012	1152 (25.2)	15 (0.3)
IMD score, median (IQR)	19.1 (11.0–34.2)	18.1 (11.0–30.4)	0.010	18.5 (11.0–31.8)	264 (5.8)
BMI, median (IQR), kg/ m^2^	27.3 (24.8–30.9)	28.2 (25.3–31.7)	<0.001	27.8 (25.1–31.3)	1794 (39.3)
Ex/current smoking status, n (%)	1251 (67.4)	1761 (64.9)	<0.001	3012 (65.9)	143 (3.1)
Caucasian, n (%)	1415 (76.2)	2071 (76.3)	0.218	3486 (76.3)	1004 (22.0)
Family history of CHD, n (%)	696 (37.5)	998 (36.8)	0.295	1694 (37.1)	599 (13.1)
Comorbidities					
Previous PCI, n (%)	68 (3.7)	245 (9.0)	<0.001	313 (6.9)	170 (3.7)
Previous CABG surgery, n (%)	39 (2.1)	251 (9.3)	<0.001	290 (6.4)	168 (3.7)
Previous AMI, n (%)	127 (6.8)	506 (18.6)	<0.001	633 (13.9)	169 (3.7)
Previous angina, n (%)	162 (8.7)	734 (27.0)	<0.001	896 (19.6)	169 (3.7)
Chronic renal failure, n (%)	17 (0.9)	106 (3.9)	<0.001	123 (2.69%)	180 (3.9)
Hypertension, n (%)	644 (34.7)	1306 (48.1)	<0.001	1950 (42.7)	178 (3.9)
Chronic heart failure, n (%)	6 (0.3)	72 (2.7)	<0.001	78 (1.71)	177 (3.9)
Hypercholesterolaemia, n (%)	469 (25.3)	927 (34.2)	<0.001	1396 (30.6)	216 (4.7)
Peripheral vascular disease, n (%)	43 (2.3)	109 (4.0)	0.002	152 (3.3)	302 (6.6)
Asthma/COPD, n (%)	184 (9.9)	370 (13.6)	0.001	554 (12.1)	188 (4.1)
Cerebrovascular disease, n (%)	52 (2.8)	134 (4.9)	0.001	186 (4.1)	180 (3.9)
Diabetes mellitus, n (%)	202 (10.9)	524 (19.3)	<0.001	726 (15.9)	93 (2.0)
Treatments during hospitalisation					
PCI, n (%)	654 (35.2)	1169 (43.1)	<0.001	1823 (39.9)	735 (16.1)
CABG surgery, n (%)	36 (1.9)	296 (10.9)	<0.001	332 (7.3)	735 (16.1)
Discharge medications					
Aspirin, n (%)	1720 (92.7)	2228 (82.1)	<0.001	3948 (86.4)	142 (3.1)
Beta blocker, n (%)	1650 (88.9)	2031 (74.8)	<0.001	3681 (80.6)	144 (3.2)
Statins, n (%)	1714 (92.4)	2245 (82.7)	<0.001	3959 (86.6)	141 (3.1)
ACEi, n (%)	1658 (89.3)	2061 (75.9)	<0.001	3719 (81.4)	161 (3.5)
Cardiac rehabilitation					
Cardiac rehabilitation offered at baseline, n (%)	1734 (93.4)	2467 (90.9)	<0.001	4201 (91.9)	264 (5.8)
Adverse cardiac events					
Deaths in hospital n (%)	2 (0.1)	14 (0.5)	0.022	16 (0.3)	61 (1.3)
Reinfarction in hospital n (%)	16 (0.9)	14 (0.5)	0.153	30 (0.7)	151 (3.3)
HRQoL					
Baseline EQ-VAS, mean (SD)	65.0 (19.8)	63.8 (20.0)	0.051	64.3 (19.9)	193 (4.2)

ACEi, ACE inhibitor; AMI, acute myocardial infarction; BMI, Body Mass Index; CABG, coronary artery bypass graft; CHD, coronary heart disease; COPD, chronic obstructive pulmonary disease; HRQoL, health-related quality of life; IMD, Index of Multiple Deprivation; NSTEMI, non-ST-elevation myocardial infarction; PCI, percutaneous coronary intervention; STEMI, ST-elevation myocardial infarction.

### HRQoL trajectories

**Figure 1:**
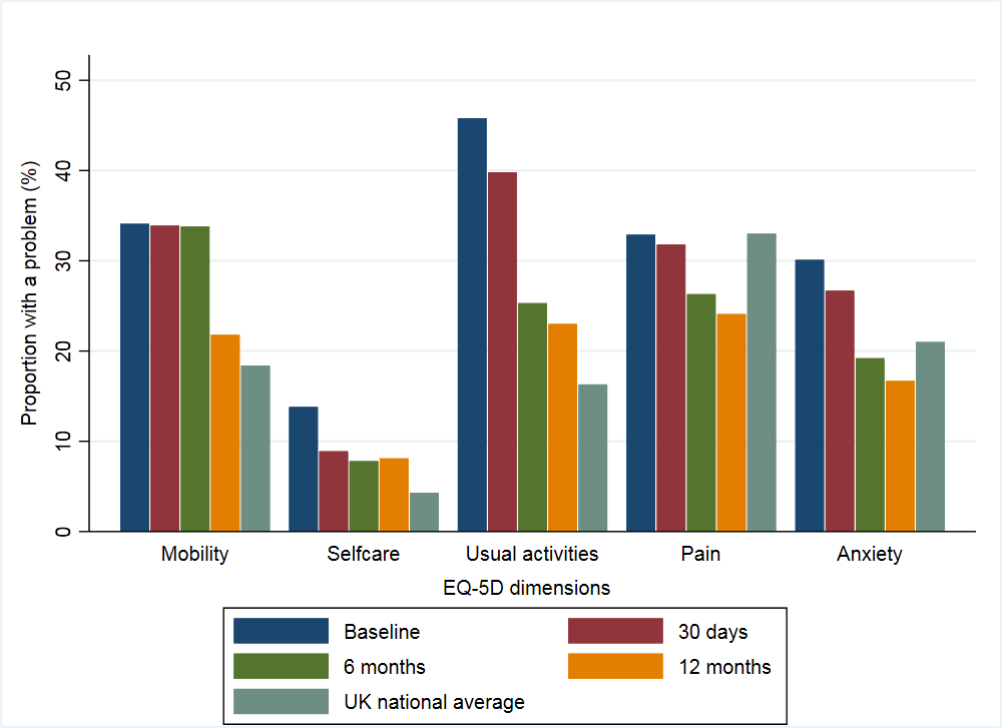
Proportion of patients with acute myocardial infarction who reported ≥1 problem across all of the EuroQol 5-Dimension domains at baseline, 30 days, and 6 and 12 months of follow-up. UK national average included.

**Figure 2:**
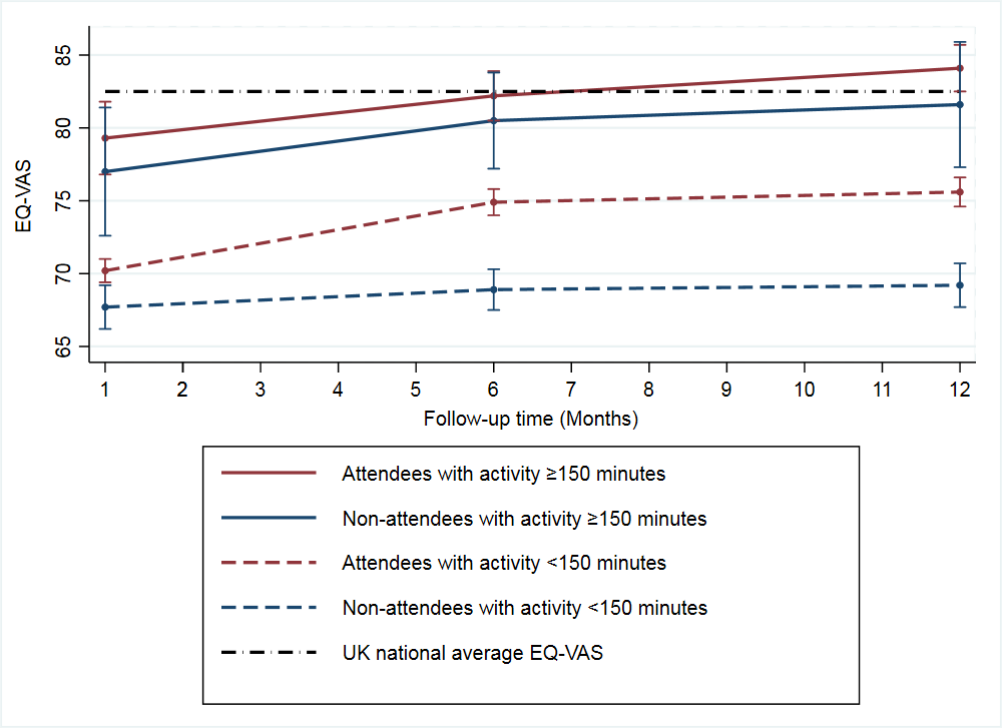
Health-related quality of life trajectories of patients with acute myocardial infarction according to their attendance at cardiac rehabilitation and/or self-reported physical activity of ≥150 min/week.

For EQ-5D dimensions, 66.6% (2893/4344) of participants reported ≥1 problem at hospitalisation, which increased to 70.8% (2356/3326) at 30 days, then decreased to 58.4% (1712/2930) at 6 months, and 56.9% (1584/2783) at 12 months. Participants reported having the greatest burden of problems (of any level) at hospitalisation in usual activities (45.8%), followed by mobility (35.5%), pain (32.9%), anxiety (30.2%) and self-care (13.8%) (figure 1 and online supplementary eTable 4). Each domain improved at each time point over the 12-month period except for self-care, which showed a modest increase from 7.7% to 8.2% at 6 and 12 months, respectively. Compared with the UK averages, participants reported a greater proportion of problems in usual activities, self-care and mobility at all time points. Anxiety was reported at a greater proportion at hospitalisation and 30 days; however, this then improved to below the UK average at 6 and 12 months. At hospitalisation, pain was reported at a similar proportion to the UK average but improved at each subsequent follow-up point. Patients with NSTEMI reported an increased frequency of ≥1 problem at all time points, at hospitalisation (65.5% vs 60.1%), 30 days (53.4% vs 48.9%), 6 months (39.9% vs 33.9) and 12 months (37.7% vs 30.2%). This pattern of improvement from hospitalisation to 12 months was also reflected in the index scores of all patients with AMI (EQ-5D-3L: 0.74 (SD 0.28) vs 0.79 (SD 0.26); EQ-VAS: 64.3 (SD 19.9) vs 74.4 (SD 18.5)) (table 2). Patients with NSTEMI reported worse HRQoL than those with STEMI at each time point except at baseline, such that the mean EQ-5D-3L scores and EQ-VAS scores were 0.74 vs 0.78 and 69.6 vs 71.2 at 30 days, 0.78 vs 0.81 and 72.9 vs 75.3 at 6 months, and 0.78 vs 0.82 and 73.1 vs 76.5 at 12 months, respectively, for NSTEMI compared with STEMI (online supplementary eTable 5). Furthermore, those who attended cardiac rehabilitation and reported physical activity of ≥150 min/week had greater temporal improvements in HRQoL scores compared with those who attended and reported physical activity of ≤150 min/week: 30 days (mean EQ-VAS: 79.3 (SD 14.6) vs 70.2 (SD 17.0)), 6 months (EQ-VAS: 82.2 (SD 13.9) vs 74.9 (SD 16.7)) and 12 months (EQ-VAS: 84.1 (SD 12.1) vs 75.6 (SD 17.0)) (figure 2 and online supplementary eTable 6).

**Table 2:** Mean EQ-5D-3L and EQ-VAS scores at baseline, 30 days, and 6 and 12 months of patients attending cardiac rehabilitation versus those who did not attend

Variable	Attended cardiac rehabilitation	Did not attend cardiac rehabilitation	P value	All AMI, n=4570	Missing, n (%)
EQ-5D Index score, mean (SD)					
Hospitalisation (SD)	0.766 (0.264), n=1681	0.754 (0.277), n=874	0.302	0.744 (0.280)	226 (5.0)
30 days, mean (SD)	0.773 (0.232), n=2259	0.728 (0.278), n=874	<0.001	0.757 (0.250)	1244 (27.2)
6 months, mean (SD)	0.821 (0.236), n=1862	0.737 (0.290), n=885	<0.001	0.790 (0.261)	1640 (35.9)
12 months, mean (SD)	0.832 (0.225), n=1725	0.739 (0.294), n=887	<0.001	0.794 (0.260)	1787 (39.1)
EQ-5D VAS score, mean (SD)					
Hospitalisation (SD)	65.4 (19.6) n=1,694	64.5 (20.0), n=880	0.312	64.3 (19.9)	193 (4.2)
30 days, mean (SD)	71.0 (16.8), n=2251	68.6 (19.7), n=865	<0.001	70.2 (17.8)	1269 (27.8)
6 months, mean (SD)	76.0 (16.4), n=1841	70.2 (19.0), n=867	<0.001	73.9 (17.7)	1691 (37.0)
12 months, mean (SD)	76.9 (16.8), n=1691	70.4 (20.3), n=884	<0.001	74.4 (18.5)	1840 (40.3)

EQ-5D-3L, EuroQol 5-Dimension-3 Level Questionnaire; EQ-VAS, EuroQol Visual Analogue Scale.

### Association of cardiac rehabilitation with HRQoL trajectories

**Figure 3:**
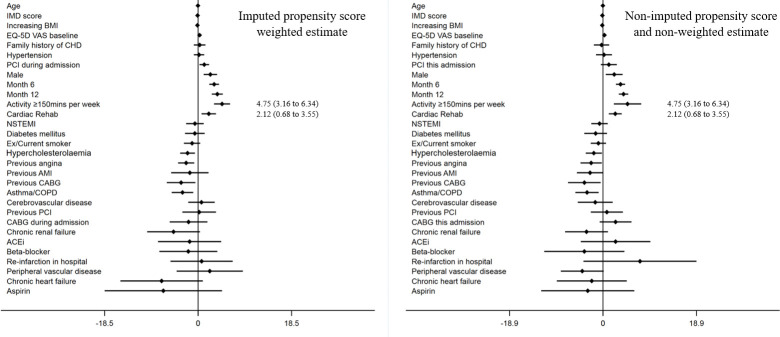
Results of the imputed propensity score weighted multi-level modelling of the association of cardiac rehabilitation and change in EQ-VAS following AMI (regression coefficients, 95% confidence intervals). ACEi, ACE inhibitor; AMI, acute myocardial infarction; BMI, Body Mass Index; CABG, coronary artery bypass graft; CHD, coronary heart disease; COPD, chronic obstructive pulmonary disease; EQ-5D VAS, EuroQol 5-dimension Visual Analogue Scale; IMD, Index of Multiple Deprivation; PCI, percutaneous coronary intervention; NSTEMI, non-ST-elevation myocardial infarction.

Participants who attended cardiac rehabilitation had higher HRQoL scores compared with those who did not attend. Moreover, attendees showed a greater temporal improvement at each follow-up point: 30 days (mean EQ-VAS: 71.0 (SD 16.8) vs 68.6 (SD 19.8)), 6 months (EQ-VAS: 76.0 (SD 16.4) vs 70.2 (SD 19.0)) and 12 months (EQ-VAS: 76.9 (SD 16.8) vs 70.4 (SD 20.4)). The propensity weighted multilevel model demonstrated temporal improvements in HRQoL following AMI over the 12-month follow-up period (EQ-VAS score coefficient at 6 months: 3.18 (95% CI 2.22 to 4.14) and at 12 months: 3.81 (95% CI 2.72 to 4.90) compared with 30 days). Cardiac rehabilitation and self-reported physical activity of ≥150 min/week were also positively associated with HRQoL (2.12 (95% CI 0.68 to 3.55) and 4.75 (95% CI 3.16 to 6.34), respectively) (figure 3). After standardisation for case mix, there was minimal variance between hospitals. However, there was wide variation between patients (48.7%) and within patients over time (51.3%) (online supplementary eTable 7).

## Discussion

In this national longitudinal study of 4570 patients hospitalised with AMI, we found that attendance at cardiac rehabilitation was associated with a temporal improvement in HRQoL at up to 12 months following hospital discharge. Moreover, for patients who also participated in activities of ≥150 min/week, the magnitude of the positive association between cardiac rehabilitation and improvements in HRQoL was larger.

It has previously been suggested that the minimal clinically important difference for HRQoL as measured by EQ-VAS is 7 points.^16^ We found that at 12 months, the absolute difference in EQ-VAS was 14.9 points when comparing patients who both attended cardiac rehabilitation and undertook additional physical activity with those who did neither (84.1 vs 69.2). Moreover, the absolute difference in EQ-VAS was 6.5 when comparing attendees to non-attendees of cardiac rehabilitation alone (76.9 vs 70.4). The propensity score wei

ghted modelling, on the other hand, found that attending cardiac rehabilitation and physical activity of ≥150 min/week at 30 days had a positive impact on changes in HRQoL (EQ-VAS score coefficient of 2.12 (95% CI 0.68 to 3.55) and 4.75 (95% CI 3.16 to 6.34)). This relates to the average relative increase over the follow-up period. Although the coefficients are not equal or greater than 7, we considered the positive impact clinically relevant.

Our results build on the existing literature. A systematic review of randomised controlled trials reported improvements in HRQoL measures in 14/20 studies for patients with AMI, previous coronary revascularisation or established CHD who participated in an exercise-based cardiac rehabilitation programme compared with those who received standard care.^4^ However, they were unable to undertake a meta-analysis due to heterogeneity in outcome measures and reporting methods. Many previous studies have also been limited by small sample sizes and non-randomisation of treatment arms or have been predominantly not of UK/European cohorts.^19–22^

Notably, the rehabilitation after myocardial infarction (randomised controlled) trial (RAMIT) found no significant effect of cardiac rehabilitation on mortality or HRQoL.^23^ Much has been debated regarding these findings particularly as the majority of the evidence for cardiac rehabilitation is from studies conducted prior to modern advancements in pharmacotherapeutic and invasive coronary strategies; it has been suggested that cardiac rehabilitation may no longer possess significant benefits for patients.^24^ However, there were a number of issues which may have contributed to the results of RAMIT, including lack of sufficient study power and a potential loss of clinical equipoise. The UK National Audit of Cardiac Rehabilitation reports that only <30% of patients offered cardiac rehabilitation declined attending, whereas in RAMIT, there was a 50% risk that participants would miss out on cardiac rehabilitation and therefore may not have participated.^25 26^

Observational longitudinal population-based studies such as ours negate some of the above issues by allowing the nesting of time periods, which enables investigation of trajectories of HRQoL and potentially within a much larger cohort of patients over time. Our data suggest that cardiac rehabilitation has an important role to play in HRQoL recovery after AMI, and encouragement of sustained physical activity beyond the initial period of cardiac rehabilitation may lead to even greater benefits in HRQoL over time.

While there are many strengths to this work, including its nationwide and longitudinal design and the efficiency of data enhancement through linkage to national clinical registries, we acknowledge the inherent weakness in the observational design. Foremost is that we do not propose a causal relationship, which can only be tested in randomised studies. Nevertheless, we undertook a weighted propensity score analysis which will, in part, have mitigated some of the confounding by indication. This analysis supports the relationship between cardiac rehabilitation and temporal improvements in HRQoL. Although we used a generic HRQoL indicator, this has previously been validated in patients post-AMI, and the domains are typically expected to be affected by AMI.^12^ There was a loss to follow-up of nearly 40% by 12 months, which is not unexpected when conducting cohort studies. This may, however, have resulted in a selection bias should those lost had worse HRQoL. Notwithstanding this, the multilevel model technique enabled inclusion of all patients in the analysis, even if they were not assessed at all follow-up points. There is also potential for recall bias or misclassification in respect to self-reported physical activity, given that patients had to report how often they were physically active for more than 15 min in a week and whether this was of light, moderate or strenuous in intensity. As with all observational studies, there is a possibility that HRQoL after AMI is affected by unmeasured confounders, which has not been accounted for in the analysis, including coronary anatomy, other comorbidities, variation between cardiac rehabilitation programmes, acuity of presentation and also acuity of intervention.

## Conclusions

This national cohort study of 4570 patients demonstrated that cardiac rehabilitation was associated with improved HRQoL. Furthermore, it suggests that these benefits were even greater for those who participated in ≥150 min of physical activity per week. Therefore, encouraging patients to participate in cardiac rehabilitation and to pursue sustained physical activity may be important for recovery post-AMI. Further studies are required to determine if the dose of exercise training and or heightened levels of physical activity status alongside cardiac rehabilitation may result in even greater benefits for patients.

**Key messages**

**What is already known on this subject?**

- Cardiac rehabilitation is associated with better health-related quality of life (HRQoL) following acute myocardial infarction (AMI).
- However, there is a paucity of information about the impact of cardiac rehabilitation on temporal changes in HRQoL in the modern pharmacotherapeutic and invasive coronary era.
- To date, there are no published nationwide longitudinal studies of repeated measures of perceived HRQoL following AMI.

**What might this study add?**

- This national longitudinal cohort study across 48 hospitals of 4570 patients with AMI found that attendance at a cardiac rehabilitation programme was independently associated with temporal improvements in HRQoL at up to 12 months following hospitalisation, with such changes further improved in patients who were physically active.

**How might this impact on clinical practice?**

- Ensuring the provision of cardiac rehabilitation services that enable all patients with AMI to attend may be an important determinant of the recovery of HRQoL.
