# Supplementary material for: Association of cardiac rehabilitation and health-related quality of life following acute myocardial infarction
Source: Heart. 2020 Aug 21;106(22):1726–31. doi: 10.1136/heartjnl-2020-316920 (PMC7656151; doi:10.1136/heartjnl-2020-316920)
Supplement: Supplementary data [file heartjnl-2020-316920supp001.pdf]

Supplementary Material

Title: Association of cardiac rehabilitation and health-related quality of life following acute myocardial infarction.

Authors: Ben Hurdus MBBS, Theresa Munyombwe PhD, Tatendashe B. Dondo PhD, Suleman Aktaa MD, Gerard Oliver, Marlous Hall PhD, Patrick Doherty PhD, Alistair S Hall PhD FRCP, Chris P. Gale PhD FRCP

eFigure 1. Derivation of the analytical cohort from the EMMACE-3 dataset.

eFigure 2: Overlap assumption assessment plots (A) and Distribution of propensity scores across treatment and comparison groups (B).

eTable 1: Covariate balance across those who attended and those who did not attend cardiac rehabilitation after weighting on the propensity score

eFigure 3: ROC curve for the propensity scoring model

eFigure 4: Normal probability plot of residuals

eTable 2: Imputation strategy

eTable 3. Comparison of baseline characteristics between respondent and non-respondents at 12 months.

eTable 4: EQ-5D-3L domain responses for patients with AMI at hospitalization, 30 days, 6 months and 12 months

eTable 5. Mean EQ-5D-3L and EQ-VAS scores at baseline, 30 days, 6 months and 12 months

eTable 6. HRQoL utilities dependent on attendance of cardiac rehabilitation and self-reported physical activity level

eTable 7: Results from the imputed and propensity score weighted multi-level modelling of association of cardiac rehabilitation on change in EQ-VAS following AMI

eReferences

Hurdus B, *et al. Heart* 2020; 106:1726–1731. doi: 10.1136/heartjnl-2020-316920

eFigure 1: Derivation of the analytical cohort from the EMMACE-3 dataset.

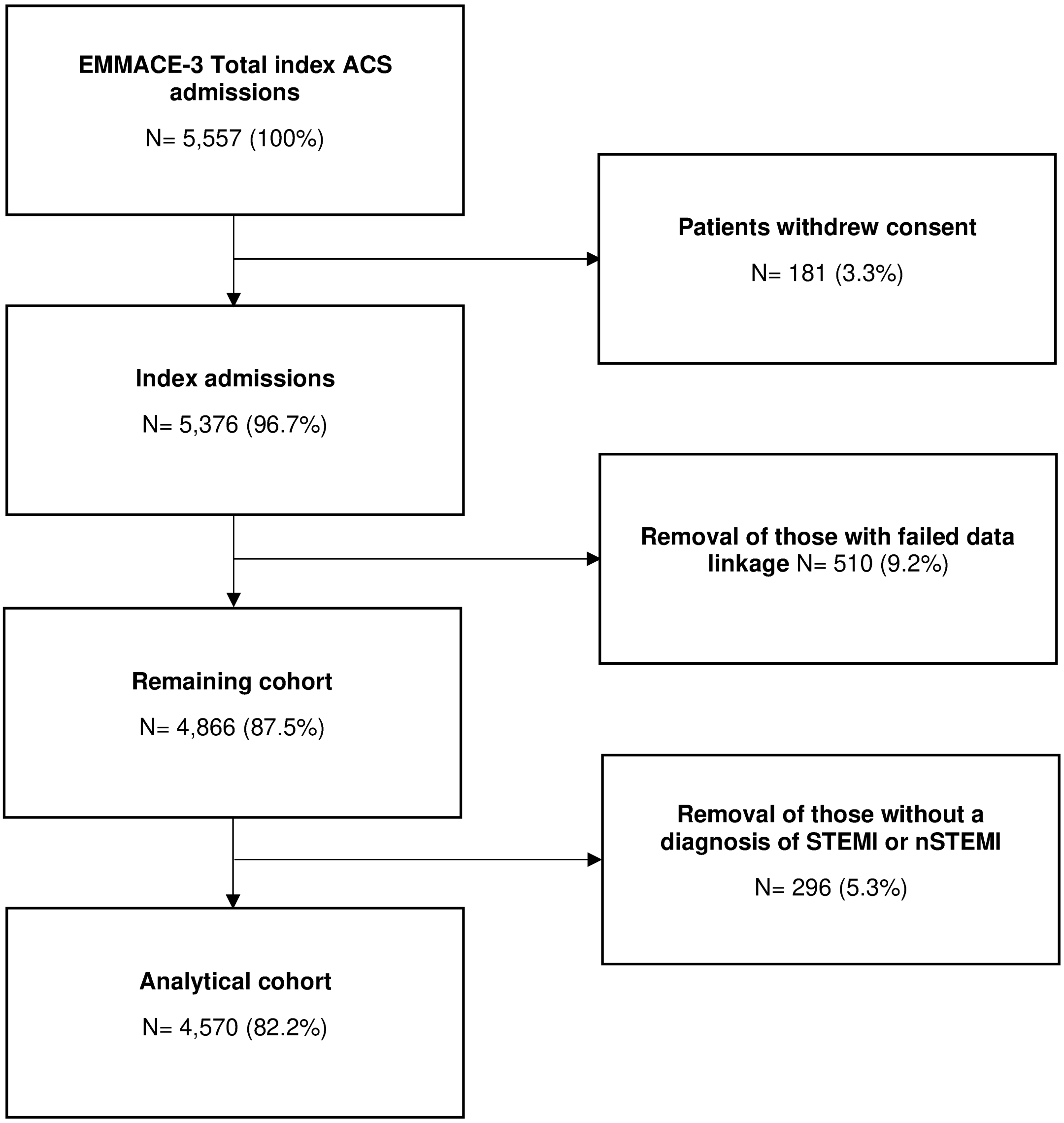

Hurdus B, *et al. Heart* 2020; 106:1726–1731. doi: 10.1136/heartjnl-2020-316920

Supplementary Methods

Section 1

Propensity score analysis

A non-parsimonious multivariable logistic regression model was used for the treatment assignment model to derive propensity scores (PS) (probability of attending cardiac rehabilitation at 30 days, conditional on observed patient baseline covariates) to weight the data. The model was adjusted for patient baseline characteristics; demographics (age, sex, body mass index, ethnicity, index of multiple deprivation [IMD]), cardiovascular risk factors (diabetes, hypercholesterolaemia, hypertension, smoking status, COPD, family history of CHD), cardiovascular history (cerebrovascular disease, peripheral vascular disease, previous angina, previous AMI, previous PCI, previous CABG), discharge medications (statins, aspirin, $P2Y_{12}$ inhibitors, angiotensin converting enzyme inhibitors [ACEi]/angiotensin receptor blockers [ARB]), coronary intervention, final diagnosis, re-infarction during index admission, baseline EQ-VAS and self-reported physical activity at 30 days. In order to assess whether the weights constructed from the treatment assignment model balanced the covariates between those who attended and those who did not attend cardiac rehabilitation , standardised differences were derived, a perfectly balanced covariate has a standardised difference of zero. Violation of the overlap assumption was assessed using an overlap plot and by summarising the estimated probabilities of treatment assignment. Observations with estimated propensity scores outside the pre-specified range 0.1 to 0.9 were discarded [1] as including these observations would have resulted in violation of the second assumption of the propensity modelling which requires estimated propensity scores for all observations to be greater than zero and less than one to assume unconfoundedness using propensity score analysis. Eighty nine patients were excluded because they had propensity scores outside the

Hurdus B, *et al. Heart* 2020; 106:1726–1731. doi: 10.1136/heartjnl-2020-316920

pre-specified range 0.1 to 0.9. eFigure 2 illustrates the results of the assessment of the overlap assumption and shows that the minimum propensity score for each treatment level was sufficiently greater than zero and that the maximum propensity score for each treatment level was sufficiently less than 1, thus the assumption was not violated. The balance check results are summarised in eTable 1, which shows that the standardised differences for variables in the weighted data were close to zero. The diagnostic assessments suggest that weighting by the inverse probability of treatment created a sample in which the distributions of the covariates were similar between those who attended and those who did not attend cardiac rehabilitation. However, the area under the curve for the propensity score model was 0.64 (eFigure 3), which indicates moderate discrimination for the model.

Hurdus B, *et al. Heart* 2020; 106:1726–1731. doi: 10.1136/heartjnl-2020-316920

eFigure 2: Overlap assumption assessment plots (A) and distribution of propensity scores across comparison groups (B): those who attended cardiac rehabilitation vs those who did not attend cardiac rehabilitation

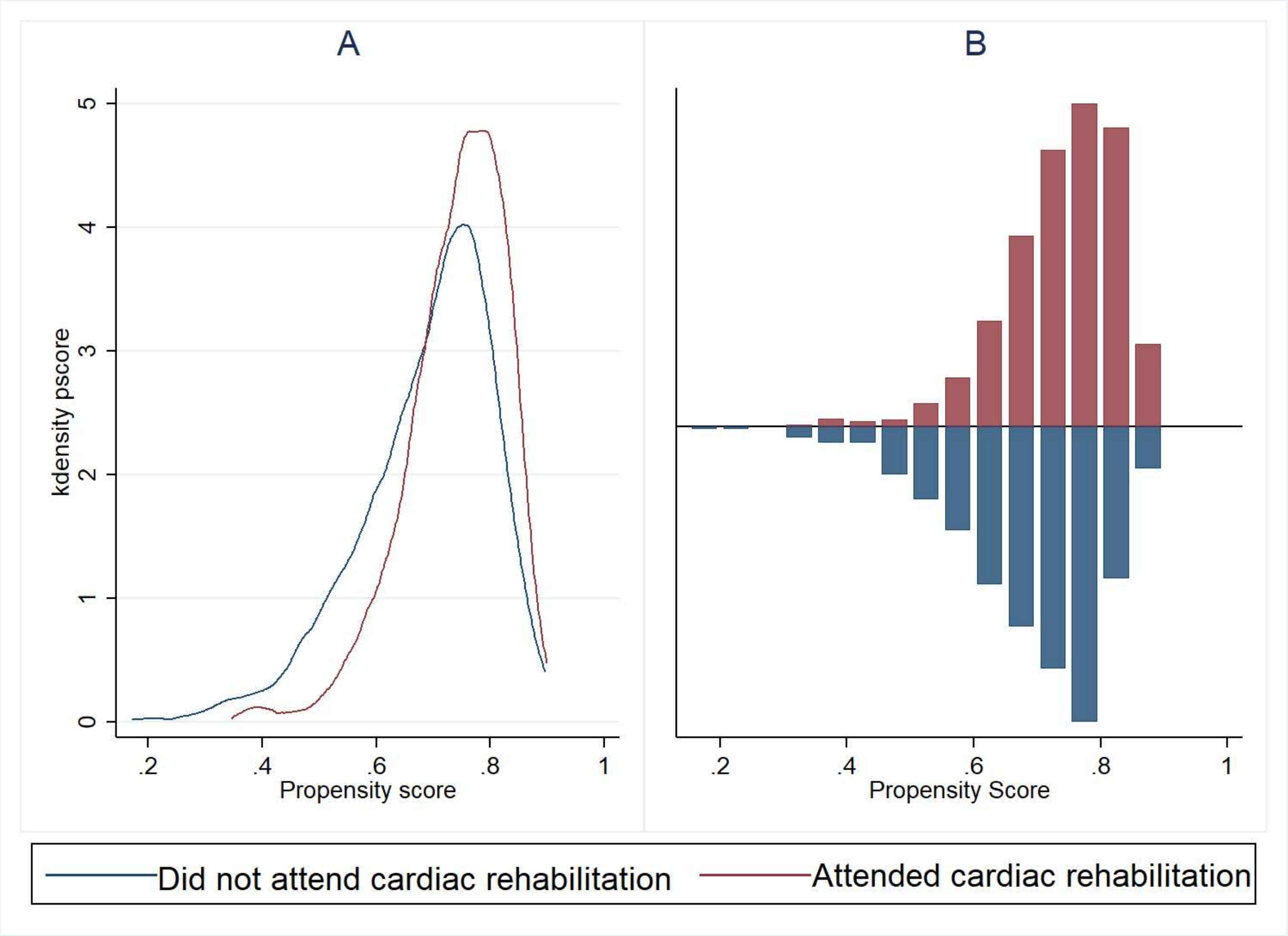

eTable 1: Covariate balance across comparison groups after weighting on the propensity score: those who attended cardiac rehabilitation vs those who did not attend cardiac rehabilitation

**Variable**	**Mean in those who attended cardiac rehabilitation**	**Mean in those who did not attend cardiac rehabilitation**	**Standardized difference**
EQ-VAS on admission	64.03	64.83	-0.041
Age	63.76	62.75	0.087
Deprivation (IMD score)	21.29	25.52	-0.276
BMI	28.19	28.73	-0.108
Smoker ever	0.66	0.66	-0.008
Ethnicity (white vs. other)	0.98	0.99	-0.051
Final diagnosis	0.43	0.40	0.048
Family history of CHD	0.41	0.39	0.043
Previous PCI	0.06	0.08	-0.048
Previous CABG	0.05	0.05	-0.005
Previous AMI	0.11	0.11	-0.002
Previous angina	0.18	0.19	-0.029
Hypertension	0.44	0.48	-0.064
Hypercholesterolaemia	0.33	0.37	-0.075
Peripheral vascular disease	0.03	0.02	0.098
Cerebrovascular disease	0.05	0.04	0.019
COPD	0.11	0.11	0.019
Chronic renal failure	0.03	0.03	0.027
Chronic cardiac failure	0.01	0.01	0.004
Diabetes	0.12	0.12	-0.015
Discharge medications			
Aspirin	0.88	0.89	-0.040
β blockers	0.83	0.84	-0.025
Statin	0.88	0.88	-0.004
ACEi/ARBs	0.84	0.85	-0.030
Coronary intervention	0.61	0.65	-0.066
Coronary re-infarction	0.07	0.03	0.057
Physical activity at 30 days (>150 mins/ week)	0.07	0.07	-0.001

Note: EQ-VAS indicates EuroQol Visual Analogue Scale; IMD, Index of Multiple Deprivation; BMI, body mass index; CHD, Coronary heart disease; PCI, percutaneous coronary intervention; CABG, coronary artery bypass graft; AMI, Acute Myocardial Infarction; COPD, chronic obstructive pulmonary disease; ACEi, Angiotensin converting enzyme inhibitor; ARB, Angiotensin receptor blocker.

Hurdus B, *et al. Heart* 2020; 106:1726–1731. doi: 10.1136/heartjnl-2020-316920

eFigure 3: ROC curve for the propensity scoring model

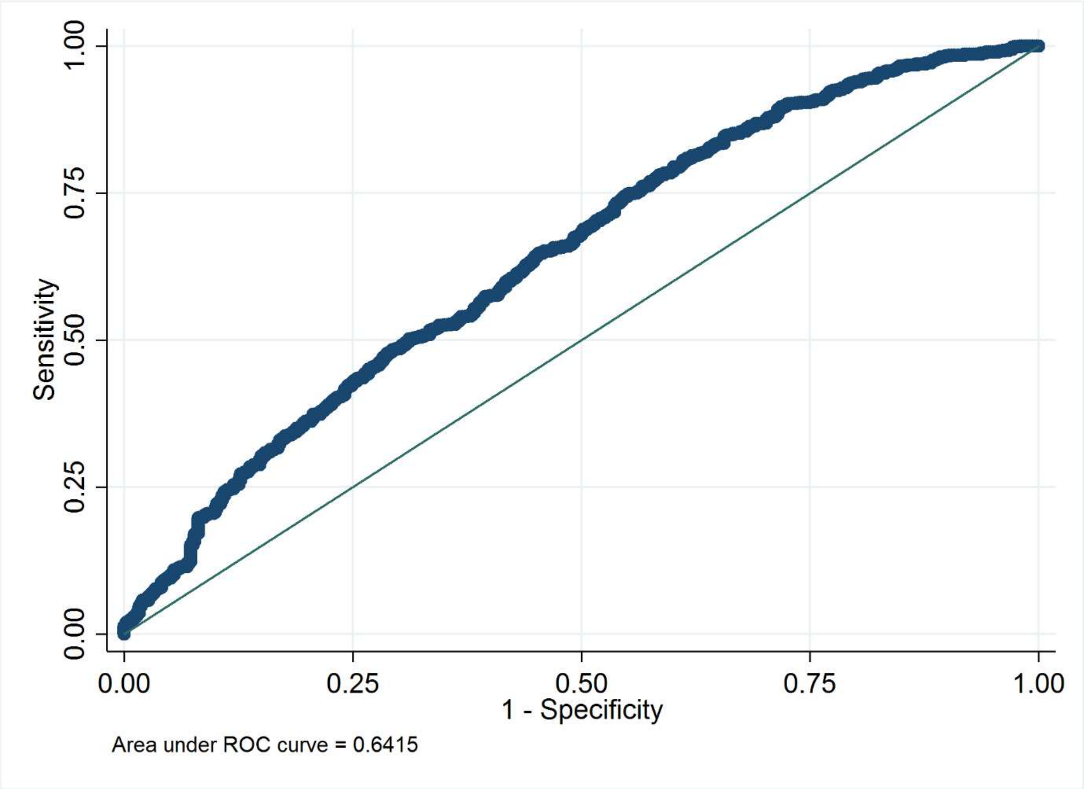

Section 2

Multi-level modelling analysis

Multi-level models [2] also known as hierarchical or mixed effects models were used to analyse the longitudinal EQ-VAS scores. Longitudinal data lacks independence because repeated measurements are nested within individuals. Ignoring the clustered nature of the data will result in underestimated standard errors of regression coefficients leading to increased type 1 errors (false positives). In this study we accounted for the clustering in the data by using multi-level models with hospital as the upper level (Level 3), patients as the second level, and repeated measurements within an individual person as the first level. Patients with less than 4 assessments were included in the analysis because multi-level models can accommodate data in which the number of timing observations vary among patients. In this study, 2032 patients had all 4 measures, 1000 had three measures, 674 had two measures, 811 had one measure, and 53 had no measures.

We adopted the approach recommended by Singer et al [3] for fitting multilevel models and considered the following three models. Model 1 was the null model partitioning between individual and within individual variation, model 2 was a random intercepts model allowing each individual to have its own intercept (initial value) but same slope (rate of change) and model 3 was a random intercept and random slope model (allowing individual initial scores and individual rate of change to vary around the mean trajectory). The most appropriate model between a random intercept and random slopes model was selected using the likelihood ratio test. Quantile-quantile plots of residuals were used to test the normality assumption for the multilevel models that were fitted, there is slight violation of the normality assumption at the tails possibly where data maybe scarce (eFigure 4).

Hurdus B, *et al. Heart* 2020; 106:1726–1731. doi: 10.1136/heartjnl-2020-316920

eFigure 4. Quantile-quantile plot of residuals

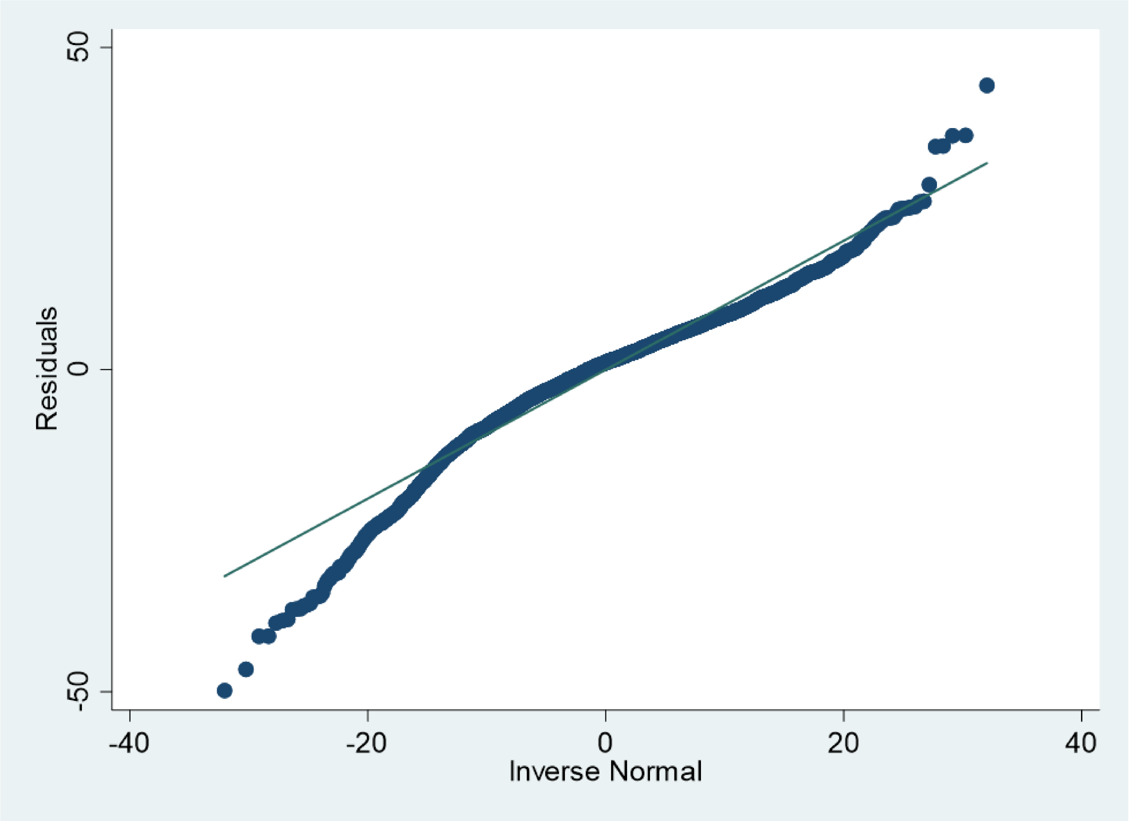

Hurdus B, *et al. Heart* 2020; 106:1726–1731. doi: 10.1136/heartjnl-2020-316920

Missingness analyses

Baseline characteristics

Missing data were imputed using multiple imputations by chained equations.[4] Ten imputed datasets were derived from 20 iterations. Data were imputed for missing baseline characteristics and not follow-up outcome data. A default imputation (missing data default imputed to "NO") strategy based on clinical expert opinion was implemented for cardiovascular history, cardiovascular risk factors, and categorical treatment variables. The imputation strategy applied is summarised in eTable 2.

eTable 2: Imputation Strategy

Variable	Variable Type	Missing (%)	Imputation method
Age	Continuous	17 (0.4)	Predictive mean matching
Deprivation (IMD score)	Continuous	264 (5.8)	Predictive mean matching
BMI	Continuous	1794 (39.3)	Predictive mean matching
Smoker ever	Binary	143 (3.1)	Predictor/ Auxiliary and Default imputed
Ethnicity (white vs. other)	Binary	1004 (22.0)	Predictor/ Auxiliary /Partially Observed
Final diagnosis	Binary	0 (0)	Predictor/ Auxiliary
Family history of CHD	Binary	599 (13.1)	Predictor/ Auxiliary and Default imputed
Previous PCI	Binary	170 (3.7)	Predictor/ Auxiliary and Default imputed
Previous CABG	Binary	168 (3.7)	Predictor/ Auxiliary and Default imputed
Previous AMI	Binary	169 (3.7)	Predictor/ Auxiliary and Default imputed

Hurdus B, *et al. Heart* 2020; 106:1726–1731. doi: 10.1136/heartjnl-2020-316920

Previous angina	Binary	169 (3.7)	Predictor/ Auxiliary and Default imputed
Hypertension	Binary	178 (3.9)	Predictor/ Auxiliary and Default imputed
Hypercholesterolaemia	Binary	216 (4.7)	Predictor/ Auxiliary and Default imputed
Peripheral vascular disease	Binary	302 (6.6)	Predictor/ Auxiliary and Default imputed
Cerebrovascular disease	Binary	180 (3.9)	Predictor/ Auxiliary and Default imputed
COPD	Binary	188 (4.1)	Predictor/ Auxiliary and Default imputed
Chronic renal failure	Binary	180 (3.9)	Predictor/ Auxiliary and Default imputed
Chronic cardiac failure	Binary	177 (3.9)	Predictor/ Auxiliary and Default imputed
Diabetes	Binary	93 (2.0)	Predictor/ Auxiliary and Default imputed
Aspirin	Categorical	142 (3.1)	Predictor/ Auxiliary and Default imputed
β blockers	Categorical	144 (3.2)	Predictor/ Auxiliary and Default imputed
Statin	Categorical	141 (3.1)	Predictor/ Auxiliary and Default imputed
ACEi/ARBs	Categorical	161 (3.5)	Predictor/ Auxiliary and Default imputed
Coronary intervention	Categorical	735 (16.1)	Predictor/ Auxiliary and Default imputed
Re-infarction in hospital	Binary	151 (3.3)	Predictor/ Auxiliary and Default imputed
EQ-5D Baseline	Continuous	226 (5.0)	Predictor/ Auxiliary /Partially Observed
EQ-VAS Baseline	Continuous	193 (4.2)	Predictor/ Auxiliary /Partially Observed
Cardiac rehabilitation offered at baseline	Categorical	264 (5.8)	Predictor/ Auxiliary and Default imputed

Abbreviation : IMD indicates Index of Multiple Deprivation; BMI, body mass index; CHD, Coronary heart disease; PCI, percutaneous coronary intervention; CABG, coronary artery bypass graft; AMI, Acute Myocardial Infarction; COPD, chronic obstructive pulmonary disease; *ACEi, Angiotensin converting enzyme inhibitor; ARB, Angiotensin receptor blocker; EQ-5D, EuroQol 5-dimension; EQ-VAS, EuroQol Visual Analogue scale.*

Hurdus B, *et al. Heart* 2020; 106:1726–1731. doi: 10.1136/heartjnl-2020-316920

Follow-up data

Baseline patient characteristics were different between responders and non-responders at 12 months. Participants were classed as responders if they returned a questionnaire with a completed EQ-VAS at 30 days, 6 months or 12 months. eTable 3 provides the full details of these differences including; baseline EQ-VAS, demographics, smoking status, co-morbidities, discharge medications and re-infarction in hospital.

eTable 3: Comparison of baseline characteristics between responders and non-responders at 12 months. Participants were classed as responders if they returned a questionnaire with a completed EQ-VAS at 30 days, 6 months or 12 months.

**Variable**	**Responders n=2828 (61.9%)**	**Non-Responders n=1742 (38.1%)**	***P* value**	**Difference (95% CI)**
**Demographics**				
Age, mean (SD)	65.5 (10.8)	60.6 (12.9)	<0.001	-4.988 (-5.687 to -4.290)
Women, n (%)	715 (25.4)	437 (25.1)	0.858	-0.002 (-0.028 to 0.024)
Ethnicity (white), n (%)	2198 (98.6)	1288 (96.4)	<0.001	-0.022 (-0.033 to -0.010)
IMD score, median (IQR)	16.9 (10.4-28.9)	21.8 (12.8-35.7)	<0.001	3.763 (2.910 to 4.613)
BMI, median (IQR)	27.6 (25.0-30.9)	28.4 (25.2-32.3)	<0.001	0.746 (0.372 to 1.116)
Ex/current smoking status, n (%)	1744 (63.8)	1268 (74.9)	<0.001	0.112 (0.084 to 0.139)
STEMI, n (%)	1121 (39.6)	735 (42.2)	0.088	0.026 (-0.004 to 0.055)
Family history of CHD, n (%)	1005 (37.4)	689 (41.2)	0.019	0.038 (0.006 to 0.069)
**Comorbidities**				
Previous PCI, n (%)	197 (7.3)	116 (6.9)	0.623	-0.004 (-0.019 to 0.012)
Previous CABG, n (%)	193 (7.1)	97 (5.7)	0.075	-0.014 (-0.028 to 0.001)
Previous AMI, n (%)	374 (13.7)	259 (15.3)	0.145	0.016 (-0.006 to 0.0374)
Previous angina, n (%)	549 (20.1)	347 (20.5)	0.749	0.004 (-0.021 to 0.029)
Chronic renal failure, n (%)	68 (2.5)	55 (3.3)	0.139	0.008 (-0.003 to 0.018)

Hurdus B, *et al. Heart* 2020; 106:1726–1731. doi: 10.1136/heartjnl-2020-316920

Hypertension, n (%)	1225 (44.9)	725 (42.8)	0.201	-0.020 (-0.050 to 0.010)
Chronic heart failure, n (%)	38 (1.4)	40 (2.4)	0.018	0.010 (0.001 to 0.018)
Hypercholesterolemia, n (%)	881 (32.4)	515 (30.4)	0.216	-0.018 (-0.046 to 0.010)
Peripheral vascular disease, n (%)	88 (3.2)	64 (3.8)	0.371	0.005 (-0.006 to 0.017)
Asthma / COPD, n (%)	328 (12.0)	226 (13.4)	0.195	0.013 (-0.007 to 0.034)
Cerebrovascular disease, n (%)	107 (3.9)	79 (4.7)	0.238	0.007 (-0.005 to 0.020)
Diabetes mellitus, n (%)	407 (14.7)	319 (18.6)	<0.001	0.039 (0.016 to 0.061)
**Treatments**				
PCI this admission, n (%)	1119 (44.6)	704 (44.8)	0.549	-0.010 (-0.042 to 0.022)
CABG this admission, n (%)	209 (8.3)	123 (7.8)	0.420	-0.008 (-0.026 to 0.011)
**Discharge medications**				
Aspirin, n (%)	2438 (86.3)	1510 (86.8)	0.819	0.002 (-0.017 to 0.021)
Beta blocker, n (%)	2295 (81.2)	1386 (79.7)	0.106	-0.019 (-0.042 to 0.004)
Statins, n (%)	2440 (86.4)	1519 (87.3)	0.455	0.007 (-0.011 to 0.026)
ACEi, n (%)	2296 (81.7)	1423 (82.0)	0.942	0.001 (-0.021 to 0.023)
**Adverse cardiac events**				
Re-infarction in hospital, n (%)	12 (0.4)	18 (1.1)	0.013	0.006 (0.001 to 0.012)
**HRQoL**				
Baseline EQ-VAS mean (SD)	64.9 (19.7)	63.2 (20.3)	0.005	-1.751 (-2.971 to -0.531)

Note: IMD indicates Index of Multiple Deprivation; BMI, body mass index; CHD, Coronary heart disease; PCI, percutaneous coronary intervention; CABG, coronary artery bypass graft; AMI, Acute Myocardial Infarction; COPD, chronic obstructive pulmonary disease; STEMI, ST-elevation myocardial infarction; NSTEMI, non ST-elevation myocardial infarction; ACEi, angiotensin converting enzyme inhibitor; HRQoL, Health-related quality of life.

eTable 4 EQ-5D-3L domain responses for patients with AMI at hospitalization, 30 days, 6 months and 12 months

	**Baseline n (%)**	**30 days n (%)**	**6 months n (%)**	**12 months n (%)**
**EQ-5D Mobility, n (%)**	**n=4400**	**n=3387**	**n=2965**	**n=2822**
No problem	2840 (64.6)	2240 (66.1)	1962 (66.2)	1823 (64.6)
Some problem	1390 (30.4)	1144 (33.8)	1000 (33.7)	993 (21.7)
Severe problem	170 (3.72)	3 (0.1)	3 (0.1)	6 (0.1)
Missing	170 (3.7)	1183 (25.9)	1605 (35.1)	1748 (38.3)
**EQ-5D Self-care, n (%)**	**n=4388**	**n=3382**	**n=2961**	**n=2820**
No problem	3786 (86.3)	2976 (88.0)	2608 (88.1)	2445 (86.7)
Some problem	560 (12.8)	398 (8.7)	347 (7.6)	369 (8.0)
Severe problem	42 (1.0)	8 (0.2)	6 (0.2)	6 (0.1)
Missing	182 (4.0)	1188 (26.0)	1609 (35.2)	1750 (38.3)
**EQ-5D Usual activities, n (%)**	**n=4376**	**n=3373**	**n=2964**	**n=2823**
No problem	2283 (50.0)	1557 (34.1)	1809 (61.0)	1773 (38.8)
Some problem	1476 (32.3)	1556 (34.1)	1032 (22.6)	946 (20.7)
Severe problem	617 (13.5)	260 (5.7)	123 (2.7)	104 (2.3)
Missing	194 (4.3)	1197 (26.2)	1606 (35.1)	1747(38.2)
**EQ-5D Pain, n (%)**	**n=4393**	**n=3376**	**n=2962**	**n=2805**
No problem	2889 (63.2)	1922 (42.1)	1760 (38.5)	1705 (37.3)
Some problem	1362 (29.8)	1324 (29.0)	1069 (23.4)	977 (21.4)
Severe problem	142 (3.1)	130 (2.8)	133 (2.9)	123 (2.7)
Missing	177 (3.9)	1194 (26.1)	1608 (35.2)	1765 (38.6)
**EQ-5D Anxiety and depression, n (%)**	**n=4392**	**n=3382**	**n=2959**	**n=2819**
No problem	3014 (66.0)	2164 (47.4)	2080 (45.5)	2056 (45.0)
Some problem	1281 (28.0)	1137 (24.9)	800 (17.5)	694 (15.2)
Severe problem	97 (2.1)	81 (1.8)	79 (1.7)	69 (1.5)
Missing	178 (3.9)	1188 (26.0)	1611 (35.3)	1751 (38.3)

Note: EQ-5D indicates EuroQol-5 Dimension-3 Level (EQ-5D-3L)

Hurdus B, *et al. Heart* 2020; 106:1726–1731. doi: 10.1136/heartjnl-2020-316920

eTable 5: Mean EQ-5D-3L and EQ-VAS scores at baseline, 30 days, 6 months and 12 months stratified by type of acute myocardial infarction (STEMI vs. NSTEMI)

**Variable**	**STEMI**<br>n=1856	**NSTEMI**<br>n=2714	***P* value**	**All AMI**<br>n=4570	**Missing, n (%)**
**EQ-5D index, mean (SD)**					
Baseline, mean (SD)	0.755 (0.28)	0.737 (0.28)	0.043	0.740 (0.28)	226 (5.0)
30 days, mean (SD)	0.776 (0.24)	0.745 (0.25)	<0.001	0.757 (0.25)	1244 (27.2)
6 months, mean (SD)	0.809 (0.26)	0.777 (0.26)	<0.001	0.790 (0.26)	1640 (35.9)
12 months, mean (SD)	0.820 (0.25)	0.777 (0.27)	<0.001	0.794 (0.26)	1787 (39.1)
**EQ-5D VAS score, mean (SD)**					
Baseline, mean (SD)	65.0 (19.8)	63.8 (20.0)	0.051	64.3 (19.9)	193 (4.2)
30 days, mean (SD)	71.2 (18.0)	69.6 (17.7)	0.010	70.2 (17.8)	1269 (27.8)
6 months, mean (SD)	75.3 (17.4)	72.9 (17.9)	<0.001	73.9 (17.7)	1691 (37.0)
12 months, mean (SD)	76.5 (17.6)	73.1 (18.9)	<0.001	74.4 (18.5)	1840 (40.3)

Note: EQ-5D indicates EuroQol-5 Dimension-3 Level (EQ-5D-3L)

Hurdus B, *et al. Heart* 2020; 106:1726–1731. doi: 10.1136/heartjnl-2020-316920

eTable 6: HRQoL utilities dependent on attendance of cardiac rehabilitation and self-reported physical activity.

	Baseline	30 days (95% CI)	6 months (95% CI)	12 months (95% CI)
**Cardiac rehabilitation (yes)**				
Utilities				
EQ-VAS, mean (SD)	65.4 (19.6)	71.0 (16.8)	76.0 (16.4)	76.9 (16.8)
EQ5D, mean (SD)	0.766 (0.264)	0.773 (0.232)	0.821 (0.236)	0.832 (0.225)
physical activity intensity				
EQ-VAS if CR and ≥150mins, mean (SD)	71.0 (18.8)	79.3 (14.6)	82.2 (13.9)	84.1 (12.1)
EQ-VAS if CR and <150mins, mean (SD)	63.9 (19.8)	70.2 (17.0)	74.9 (16.7)	75.6 (17.0)
EQ5D if CR and ≥150mins, mean (SD)	0.830 (0.251)	0.859 (0.194)	0.888 (0.176)	0.913 (0.156)
EQ5D if CR and <150mins, mean (SD)	0.752 (0.266)	0.765 (0.236)	0.808 (0.245)	0.820 (0.229)
**Cardiac rehabilitation (No)**				
Utilities				
EQ-VAS, mean (SD)	64.5 (20.0)	68.6 (19.8)	70.2 (19.0)	70.4 (20.4)
EQ5D, mean (SD)	0.754 (0.277)	0.728 (0.278)	0.737 (0.290)	0.739 (0.294)
physical activity intensity				
EQ-VAS if no CR and ≥150mins, mean (SD)	67.4 (20.5)	77.0 (15.8)	80.5 (15.0)	81.6 (17.2)
EQ-VAS if no CR and <150mins, mean (SD)	63.4 (19.7)	67.7 (20.0)	68.9 (19.1)	69.2 (20.6)
EQ5D if no CR and ≥150mins, mean (SD)	0.804 (0.267)	0.868 (0.150)	0.829 (0.238)	0.875 (0.191)
EQ5D if no CR and <150mins, mean (SD)	0.736 (0.285)	0.716 (0.287)	0.731 (0.290)	0.719 (0.303)
**All patients**				
EQ-VAS, mean (SD)	64.3 (19.9)	70.2 (17.8)	73.9 (17.7)	74.4 (18.5)
EQ5D, mean (SD)	0.744 (0.280)	0.757 (0.250)	0.790 (0.261)	0.794 (0.260)
Physical activity intensity				
EQ-VAS if <150mins, mean (SD)	64.4 (19.7)	69.3 (18.0)	72.6 (18.1)	73.1 (28.8)
EQ-VAS if ≥150mins, mean (SD)	68.3 (19.3)	78.6 (15.2)	81.9 (14.0)	83.5 (13.6)

Hurdus B, *et al. Heart* 2020; 106:1726–1731. doi: 10.1136/heartjnl-2020-316920

EQ5D if <150mins, mean (SD)	0.748 (0.276)	0.749 (0.255)	0.777 (0.267)	0.778 (0.267)
EQ5D if ≥150mins, mean (SD)	0.811 (0.250)	0.859 (0.186)	0.874 (0.193)	0.903 (0.172)

Note: EQ-5D indicates EuroQol-5 Dimension-3 Level (EQ-5D-3L)

Hurdus B, *et al. Heart* 2020; 106:1726–1731. doi: 10.1136/heartjnl-2020-316920

eTable 7: Results from the imputed and propensity score weighted multi-level modelling of association of cardiac rehabilitation on change in EQ-VAS following AMI (regression coefficients, 95% confidence intervals)

**Variable**	**Regression coefficient (95% CI)**	***P* value**
**Cardiac rehabilitation**		
Undergone Cardiac Rehabilitation	2.12 (0.68 to 3.55)	0.004
**Physical activity**		
≥150mins per week	4.75 (3.16 to 6.34)	<0.001
**Follow-up time**		
Ref (30 day)		
6 months	3.18 (2.22 to 4.14)	<0.001
12 months	3.81 (2.72 to 4.90)	<0.001
**Baseline**		
Age	-0.3 (-0.12 to 0.06)	0.513
Men	2.43 (1.10 to 3.76)	<0.001
IMD score	-0.10 (-0.15 to -0.05)	<0.001
BMI	-0.13 (-0.27 to 0.01)	0.069
Ex/current smoker	-1.19 (-2.81 to 0.42)	0.147
NSTEMI	-0.63 (-2.37 to 1.11)	0.478
Family history of CHD	0.31 (-0.85 to 1.46)	0.604
**Co-morbidities**		
Previous PCI	0.22 (-3.12 to 3.55)	0.898
Previous CABG	-3.37 (-6.20 to -0.54)	0.020
Previous AMI	-1.64 (-5.34 to 2.06)	0.384
Previous angina	-2.37 (-3.97 to -0.76)	0.004
Chronic renal failure	-4.87 (-10.10 to 0.37)	0.068
Hypertension	0.18 (-0.84 to 1.21)	0.725
Chronic heart failure	-7.23 (-15.40 to 0.94)	0.083
Hypercholesterolemia	-2.09 (-3.55 to -0.63)	0.005
Peripheral vascular disease	-2.31 (-4.25 to 8.87)	0.491
Asthma / COPD	-3.09 (-5.23 to -0.96)	0.004
Cerebrovascular disease	0.67 (-1.95 to 3.30)	0.615
Diabetes mellitus	-0.65 (-2.59 to 1.30)	0.516
**Treatments**		
PCI during admission	1.22 (0.27 to 2.18)	0.012
CABG this admission	-1.89 (-5.63 to 1.85)	0.322

Hurdus B, *et al. Heart* 2020; 106:1726–1731. doi: 10.1136/heartjnl-2020-316920

**Discharge medications**		
Aspirin	-6.89 (-18.53 to 4.75)	0.246
Beta blocker	-1.94 (-7.68 to 3.81)	0.509
Statins	14.93 (1.25 to 28.61)	0.032
ACEi	-1.68 (-7.94 to 4.57)	0.598
**Adverse cardiac events**		
Re-infarction in hospital	0.70 (-5.47 to 6.86)	0.825
**HRQoL**		
EQ-5D VAS baseline	0.30 (0.25 to 0.35)	<0.001
**Random effects**		
Hospital	4.11 x e^{-6} (1.72 x e^{-43} to 9.80 x e^{31})	
Patient	10.52 (7.34 to 15.07)	
Residual	11.08 (10.13 to 12.11)	
**Intraclass correlation coefficient (%)**		
Patient	48.7	
Residual	51.3	

Note: IMD indicates Index of Multiple Deprivation; BMI, body mass index; CHD, Coronary heart disease; PCI, percutaneous coronary intervention; CABG, coronary artery bypass graft; AMI, Acute Myocardial Infarction; COPD, chronic obstructive pulmonary disease; STEMI, ST-elevation myocardial infarction; NSTEMI, non ST-elevation myocardial infarction; ACEi, angiotensin converting enzyme inhibitor; EQ-5D, EuroQol-5 Dimension

Hurdus B, *et al. Heart* 2020; 106:1726–1731. doi: 10.1136/heartjnl-2020-316920

eReferences

1. Crump RK, Hotz VJ, Imbens GW, Mitnik OA. Dealing with limited overlap in estimation of average treatment effects. Biometrika 2009:asn055
2. Goldstein H. *Multilevel statistical models*: John Wiley & Sons, 2011.
3. Singer JM, Andrade DF. Analysis of longitudinal data. Handbook of statistics 2000;**18**:115-60
4. Cattle B, Baxter P, Greenwood D, Gale C, West R. Multiple imputation for completion of a national clinical audit dataset. Stat Med 2011;**30**(22):2736-53

Hurdus B, *et al. Heart* 2020; 106:1726–1731. doi: 10.1136/heartjnl-2020-316920
